# Serum Urotensin II Levels Are Elevated in Patients with Obstructive Sleep Apnea

**DOI:** 10.3390/biom13060914

**Published:** 2023-05-31

**Authors:** Ante Mihovilovic, Zoran Dogas, Dinko Martinovic, Daria Tokic, Ema Puizina Mladinic, Marko Kumric, Natalija Ivkovic, Marino Vilovic, Josko Bozic

**Affiliations:** 1 Department of Maxillofacial Surgery, University Hospital of Split, 21000 Split, Croatia; amihovilovic@kbsplit.hr (A.M.); d.m.993@hotmail.com (D.M.); epuizina@kbsplit.hr (E.P.M.); 2 Department of Neuroscience and Sleep Medicine Center, University of Split School of Medicine, 21000 Split, Croatia; zdogas@mefst.hr (Z.D.); nivkovic96@gmail.com (N.I.); 3 Department of Anesthesiology and Intensive Care, University Hospital of Split, 21000 Split, Croatia; dtokic@kbsplit.hr; 4 Department of Pathophysiology, University of Split School of Medicine, 21000 Split, Croatia; marko.kumric@mefst.hr (M.K.); marino.vilovic@mefst.hr (M.V.)

**Keywords:** obstructive sleep apnea, urotensin II, inflammation

## Abstract

Obstructive sleep apnea (OSA) has become major public concern and is continuously investigated in new aspects of pathophysiology and management. Urotensin II (UII) is a powerful vasoconstrictor with a role in cardiovascular diseases. The main goal of this study was to evaluate serum UII levels in OSA patients and matched controls. A total of 89 OSA patients and 89 controls were consecutively enrolled. A medical history review and physical examination of the participants was conducted, with polysomnography performed in the investigated group. UII levels and other biochemical parameters were assessed according to the standard laboratory protocols. The median AHI in the OSA group was 39.0 (31.4–55.2) events/h, and they had higher levels of hsCRP when compared to control group (2.87 ± 0.71 vs. 1.52 ± 0.68 mg/L; *p* < 0.001). Additionally, serum UII levels were significantly higher in the OSA group (3.41 ± 1.72 vs. 2.18 ± 1.36 ng/mL; *p* < 0.001), while positive correlation was found between UII levels and hsCRP (r = 0.450; *p* < 0.001) and systolic blood pressure (SPB) (r = 0.317; *p* < 0.001). Finally, multiple regression analysis showed significant association of UII levels with AHI (0.017 ± 0.006, *p* = 0.013), SBP (0.052 ± 0.008, *p* < 0.001) and hsCRP (0.538 ± 0.164, *p* = 0.001). As UII levels were associated with blood pressure and markers of inflammation and OSA severity, it might play an important role in the complex pathophysiology of OSA and its cardiometabolic complications.

## 1. Introduction

Obstructive sleep apnea (OSA) is a complex pathophysiological phenomenon occurring when the upper airway is collapsed, partially or completely, causing a decrease in oxygen saturation and disturbance of the sleep [1]. There are several known factors for developing OSA and most of them can be categorized into two large groups: anatomic and non-anatomic [2].

Considering anatomic factors, some are congenital (bone alterations such as micrognathia, retrognathia or mandibular hypoplasia) while others are iatrogenic after tissue manipulation, for instance after trauma or cancer treatment [3]. When it comes to non-anatomic factors, the most common are male sex, obesity and age [4,5]. Moreover, OSA is not only a sleep and breathing related disorder, but it is also associated with several serious illnesses such as diabetes, hypertension, coronary artery disease, stroke, and overall higher mortality [6,7,8,9]. With increasing prevalence and public health impact, OSA is continuously investigated in order to find important new aspects in the pathophysiology, management and treatment of this chronic disorder.

Initially isolated from the fish spinal cord, urotensin II (UII) is a powerful vasoconstrictor, known for its significant role in regulation of the cardiovascular system [10,11]. Besides being expressed in the cardiovascular system, UII is also expressed in several other tissues in the human body, such as the nervous system, spleen, kidneys, small intestines, adrenal and pituitary glands [12]. Moreover, UII circulates in human plasma, where its levels are elevated in conditions such as kidney failure, diabetes, heart failure and hypertension [10,13]. Several studies investigated UII and its role in cardiovascular diseases and showed that levels of UII are elevated according to levels of atherosclerosis [14,15]. UII activity is regulated by the urotensin receptor (UTR) which after stimulation causes calcium mobilization in the cellular plasma, smooth muscle cells proliferation and collagen production [16]. Furthermore, several recent studies have suggested that UII could possibly also have an immunomodulatory effect, as it was shown that UII plays a role in the regulation of the inflammation process [17,18,19].

The main cardiovascular disorder related to OSA is drug-resistant systemic hypertension which shows a prevalence higher than 50% in those patients [20]. Several potential pathophysiological mechanisms have been proposed to explain this association; however, they all still leave unanswered questions regarding the link between these two disorders. Since UII is the strongest known vasoconstrictor, associated with numerous disorders related to hypertension, it is only reasonable to presume a possible connection between UII and OSA. Through extensive research of the available literature, it was found that there are no previous studies which investigated this possible link.

Hence, the aim of this study was to evaluate serum UII levels in patients with OSA and to compare them with healthy, gender- and age-matched controls. Moreover, an additional goal was to evaluate possible associations of serum UII levels with clinical, laboratory and anthropometric parameters in patients with OSA.

## 2. Materials and Methods

### 2.1. Study Design

This cross-sectional study was performed at the Sleep Medicine Center, University Hospital of Split and University of Split School of Medicine during the period between April 2018 and April 2020.

All of the participants were informed about the procedures and purpose of this study and they all signed a written consent prior to inclusion. This study was approved by the Ethics Committee of the University of Split School of Medicine (Date: 30 May 2014; No: 003-08/14-03/0003) and it was performed according to the latest revision of the Helsinki Declaration.

### 2.2. Subjects

There were 124 subjects screened for inclusion in this study. All of them had newly diagnosed OSA and they were enrolled consecutively at the Sleep Medicine Center. OSA was determined according to the most recent and relevant clinical guidelines for diagnosing OSA in adults [21]. In addition to newly diagnosed OSA, another inclusion criterion was age between the limits of 18 and 65 years. Exclusion criteria were diabetes mellitus; severe cardiovascular, neurological, psychiatric, respiratory or renal diseases; systemic autoimmune or inflammatory diseases; acute or chronic immunocompromised states; active malignancy; sedatives or narcotics usage; alcohol and drug abuse; and medical history of OSA treatment prior to this study. After the screening, 35 subjects did not satisfy the criteria; hence, the study included 89 subjects with newly diagnosed OSA.

The control group consisted of age-, gender- and body mass index (BMI)-matched volunteers, which were recruited through family, friends, colleagues and acquaintances. Physical examination and medical history inspection were conducted on 132 healthy volunteers, 89 of whom were included in the study as controls after applying the exclusion criteria and obtaining the results from sleep-related questionnaires. The STOP-Bang questionnaire, which screens 4 self-reported (snoring, tiredness, observed apnea and blood pressure) and 4 anthropometric (BMI, age, neck circumference and gender) items, was the main screening tool for identification of control subjects who have a high risk of OSA development [22]. Hence, all potential subjects who scored ≥2 points on the STOP-Bang questionnaire were excluded. Moreover, for the assessment of daytime sleepiness in common daily situations the Epworth Sleepiness Scale (ESS) was used, validated in Croatian language, and all subjects who had an ESS score >9 were excluded [23,24]. Polysomnography (PSG) assessment was not performed for the control subjects. The control group had the same exclusion criteria and assessment protocol as the OSA group.

After detailed medical history inspection and meticulous physical examination, anthropometric measurements were conducted for all the included participants. A calibrated scale was used for body mass and height measurements (Seca, Birmingham, UK). Afterwards, BMI was calculated using the formula: body mass (kg)/height squared (m^2^). For arterial blood pressure measurements, the subjects were seated for 10 min after which a sphygmomanometer was used for three consecutive measurements and the mean value was calculated.

### 2.3. Sleep Assessment

All of the included patients with OSA were submitted to a full-night PSG at the Sleep Medicine Center during which the following measurements were continuously recorded: electrooculography, electroencephalography, mental and tibial electromyography, electrocardiography, nasal airflow, pulse oximetry, thoracic and abdominal movements and snoring intensity (Alice 5LE, Philips Respironics, Eindhoven, The Netherlands). All of the collected data were stored on a computer and later manual analyses were conducted in accordance with the published American Academy of Sleep Medicine and European Sleep Research Society guidelines [25].

In accordance with the recommendations, apnea was identified as a complete cessation of airflow for at least 10 s. On the other hand, hypopnea was defined as an airflow reduction by more than 50% for at least 10 s, in combination with a hemoglobin oxygen saturation decrease of at least 3% [25]. All full-night PSG measurements which lasted less than 6 h were deemed invalid, and in these cases another sleep analysis was undertaken.

Furthermore, all of the included patients had their apnea–hypopnea index (AHI) and oxygen desaturation index (ODI) estimated. AHI is a well-established index that represents the severity of sleep apnea and is derived from the number of apnea and hypopnea events that occur per 1 h of sleep [26]. ODI is an index that averages the number of desaturation episodes that occur per 1 h of sleep. The desaturation episodes are defined as a decrease in the mean oxygen saturation of ≥3% (through the last 2 min) for at least 10 s [27].

### 2.4. Blood Sampling and Laboratory Analysis

All of the included patients underwent blood sampling at the scheduled check-up 7 to 14 days after the PSG was performed. None of the patients were on treatment for OSA during the time period between the PSG and the blood sampling. Venous blood samples were extracted after 10–12 h fasting via a polyethylene catheter inserted into the cubital vein. All of the blood sample analyses were conducted by the same biochemical laboratory and by the same biochemist according to the standard laboratory protocols. Additionally, the biochemist was highly experienced and was blinded to the participant’s role in the study.

Serum levels of UII were estimated using the enzyme immunoassay (EIA) kit for human UII (Phoenix Pharmaceuticals, Burlingame, CA, USA), according to the manufacturer’s instructions. The concentration of the analyzed quality control sample which arrived from the manufacturer was within predefined acceptable deviation. The linear range of the assay was 0.06–8.2 ng/mL and sensitivity was 0.06 ng/mL. CV within the probe was less than 10%, and between probes was less than 15%. Furthermore, the latex turbidimetric method was used for hsCRP (Abbott Laboratories, Chicago, IL, USA) level determination. All other parameters were determined according to the standard laboratory practice.

### 2.5. Statistical Analysis

All of the collected data were analyzed using the statistical software MedCalc for Windows^®^ (version 20.110, MedCalc Software, Ostend, Belgium) while all graphical figures were created using SigmaPlot for Windows^®^ (version 14.0, Systat Software Inc, San Jose, CA, USA). The normality of distribution was estimated using the Kolmogorov–Smirnov test. All categorical variables were presented as whole numbers and percentages, while quantitative variables were presented as mean ± standard deviation if normal data distribution was shown, or median (interquartile range) if the data were not normally distributed. The comparison of categorical variables between groups was conducted using the chi-square test while the comparison between quantitative variables was conducted using Student’s *t*-test. Correlation was estimated using Pearson’s correlation method. Furthermore, multiple linear regression analysis was conducted with serum UII level as a dependent variable. The independent variables were presented with their unstandardized coefficient β, standard error, *t*-value and respective *p*-value. The level of statistical significance was set at *p* < 0.05.

## 3. Results

The OSA group had a significantly higher neck circumference compared to the control group (*p* < 0.001) (Table 1). There were no other significant differences between the OSA group and the control group regarding the anthropometric parameters (Table 1). However, regarding the laboratory parameters, there was a statistically significant difference in hsCRP levels, as the OSA group had a higher level compared to the control group (2.87 ± 0.71 vs. 1.52 ± 0.68 mg/L; *p* < 0.001) (Table 1).

The median AHI in the OSA group was 39.0 (31.4–55.2) events per hour while the median ODI was 36.8 (27.8–56.1) events per hour (Table 2). The mean SpO_2_ was 94.0% (92.3–95.0%) while the lowest SpO_2_ was 76.4% (71.0–83.7%). Other PSG parameters are stated in Table 2.

**Figure 1:**
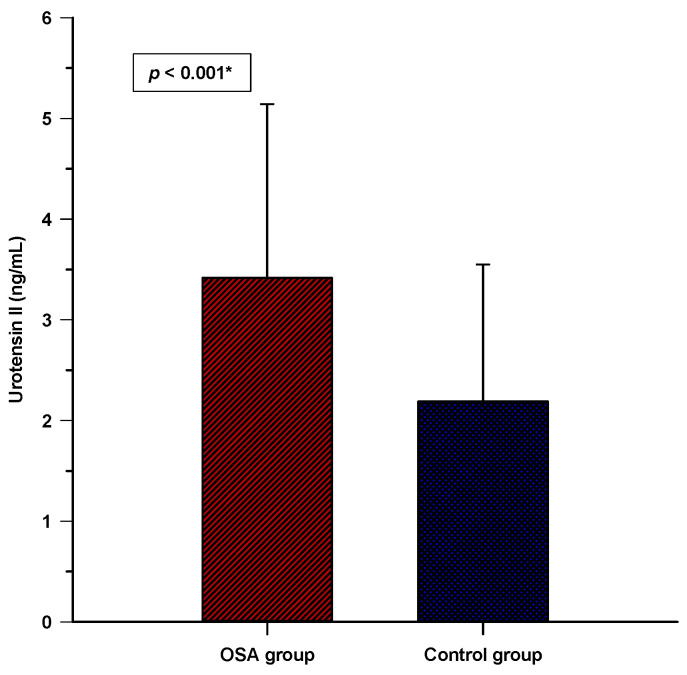
Comparison of the serum urotensin II levels between the OSA group (N = 89) and the control group (N = 89). Abbreviation: OSA—obstructive sleep apnea. Data are plotted as mean ± standard deviation. * Student’s *t*-test.

There was a statistically significant difference found in serum urotensin II levels as the OSA group had a higher serum level compared to the control group (3.41 ± 1.72 vs. 2.18 ± 1.36 ng/mL; *p* < 0.001) (Figure 1).

**Figure 2:**
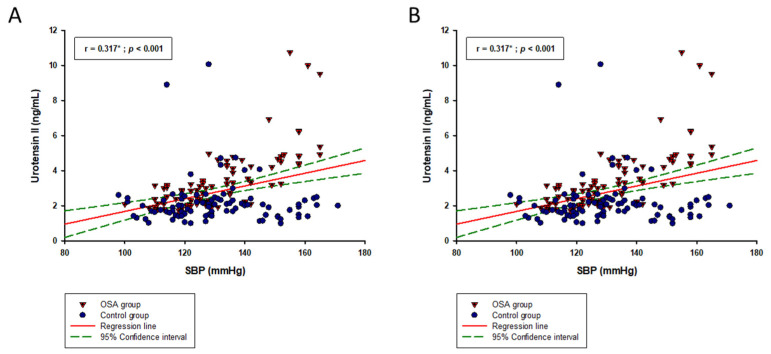
Urotensin II correlation with (**A**) SBP and (**B**) DBP indices in the whole study sample (N = 178). Abbreviations: OSA—obstructive sleep apnea; SBP—systolic blood pressure; DBP—diastolic blood pressure. * Pearson’s correlation coefficient.

**Figure 3:**
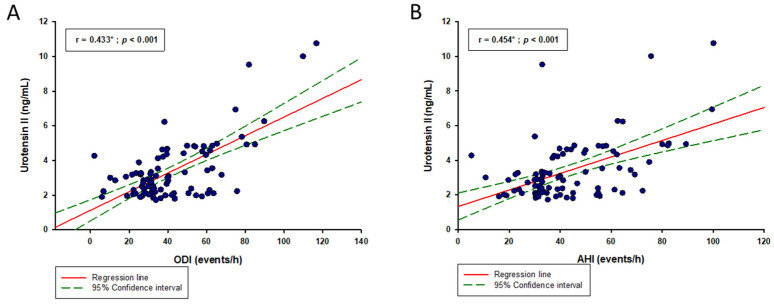
Urotensin II correlation with (**A**) ODI and (**B**) AHI indices in the OSA group (N = 89). Abbreviations: OSA—obstructive sleep apnea; AHI—apnea/hypopnea index; ODI—oxygen desaturation index. * Pearson’s correlation coefficient.

In the whole study sample, serum UII levels showed a significant, but weak positive correlation with BMI (r = 0.176; *p* = 0.019), while also a moderate positive correlation was found with hsCRP (r = 0.450; *p* < 0.001), SBP (r = 0.317; *p* < 0.001) and DBP (r = 0.379; *p* < 0.001) (Table 3) (Figure 2A,B). Likewise, in the OSA group, serum UII levels had a significant moderate positive correlation with hsCRP levels (r = 0.413; *p* < 0.001) (Table 3), while it also showed a moderate positive correlation with ODI (r = 0.433; *p* < 0.001) and AHI (r = 0.454; *p* < 0.001) indices (Figure 3A,B). On the other hand, in the control group, there was only a significant positive correlation between the UII levels and hsCRP levels (r = 0.481; *p* < 0.001).

A multiple linear regression model of the total study sample (N = 178) was calculated with serum UII levels as a dependent variable and age, BMI, SBP, DBP, hsCRP and AHI as independent variables (Table 4). ODI was not included in the model due to the high collinearity of AHI and ODI. The model showed that SBP (0.052 ± 0.008, *p* < 0.001), hsCRP (0.538 ± 0.164, *p* = 0.001) and AHI (0.017 ± 0.006, *p* = 0.013) are positive predictors of the serum UII levels (Table 4).

## 4. Discussion

The results of this study showed that serum UII levels are significantly higher in patients with OSA compared to the healthy, age- and gender-matched controls. Furthermore, there was a significant positive correlation between serum UII levels with both ODI and AHI indices. Additionally, UII levels had a significant positive correlation with hsCRP and both systolic and diastolic blood pressures. To the best of our knowledge, this is the first study which investigated serum UII levels in patients with OSA.

Several studies have attempted to define the circulating levels of UII in healthy subjects in comparison to patients with different diseases. These studies showed great discrepancy in circulating UII concentrations with values ranging from 6 pg/mL to 3.6 ng/mL [28,29]. The variations in UII serum levels in different pathologic conditions can be explained and is to be expected, but diversity among healthy subjects is confusing. The possible explanation could lie in two commonly used assay formats, radioimmunoassay (RIA) and enzyme-linked immunosorbent assay (ELISA), but only some of the antibodies used in these techniques can recognize mature UII protein and its precursor protein or various truncated UII fragments [30]. It was shown that serum UII levels are elevated in conditions such as heart failure, renal failure, liver disease and diabetes [31]. According to the review by Svistunov et al., UII is involved in the pathophysiology of many stressful and adaptive reactions and in the development of cardiovascular pathologies [32]. It also appears that its role in biomedicine is not completely clear as a recent animal study showed that the deletion of the UTR in knockout mice does not affect basal hemodynamics in comparison to wild-type mice [33].

Furthermore, UII is significantly superior to endothelin-1 in terms of vasoconstriction effect [34]. It was also shown that angiotensin II stimulates UII expression, reactive oxygen species (ROS) production and cardiac hypertrophy [35]. Since intermittent hypoxia is a hallmark manifestation of OSA, in their study, Inamoto et al. demonstrated that hypoxia in cultured rat neonatal cardiomyocytes induces expression of angiotensin II and ROS, which mediates the induction of UII expression and subsequent cardiomyocyte hypertrophy [36]. This pathophysiological mechanism could be the possible connection for elevated UII levels and OSA comorbidities. According to Prabhakar et al., patients with AHI of 5–15 events per hour and >15 events per hour are 2–3 times more at risk for developing hypertension, which matches the results of our study and implies an important role of UII in the pathophysiology of hypertension in OSA [37]. It is important to emphasize that the association between the severity of OSA and hypertension is independent of confounding factors such as BMI, age and sex [38,39]. Additionally, the possible link between OSA and UII could lie in the sympathetic nervous system activation and the consequent blood vessels constriction and blood pressure elevation through the UII pathway. However, this issue needs to be further addressed in future studies.

The results of our study showed a significant positive correlation of UII with systolic and diastolic blood pressure in OSA patients. OSA is well known for its association with an elevated risk for hypertension and cardiovascular diseases such as ischemic heart disease, stroke, and arrhythmias, as well as metabolic disorders including diabetes mellitus, lipid profile disorders and non-alcoholic fatty liver disease [40,41]. Furthermore, it was shown that UII may play an important role in the central mechanisms of blood pressure regulation. It was implied that UII participates in the complex functional reorganization of neuronal formations in the central nervous system, which may consequently lead to the occurrence of hypertension [42]. Furthermore, a study by Alicic et al., as well as several other studies, determined a positive correlation between UII levels and blood pressure, with a vasoconstrictive effect as a probable mechanism of action [43]. It is interesting that Debiec et al., in their expression studies of the UII pathway, found that common genetic variations in the UII pathway are unlikely to play a major role in genetic regulation of blood pressure or renal function, so this conclusion needs further investigation [44].

OSA has also been considered as a low-grade chronic inflammatory disease, with OSA patients having higher circulating levels of inflammatory marker hsCRP, interleukin-6, interleukin-8, TNF-α, intercellular adhesion molecule-1 and vascular cell adhesion molecule-1 [45]. Activated inflammation pathways can be either a cause or consequence of OSA and common biomarkers that indicate systemic inflammation include CRP and TNF-α; hence, they have been widely studied in OSA [46]. It is important to highlight that systemic inflammation has been connected to hypertension, atherosclerosis and cardiovascular disease, but its role in OSA may be confused by obesity [47,48,49]. In their study, Guler et al. clearly stated that UII levels, oxidative stress and inflammation are higher in hypertensive and resistant hypertensive patients, and antioxidants and thiol levels are lower than in healthy controls [50]. This finding shows association of UII with both oxidative stress and inflammation, which is in line with our outcomes.

The correlation between UII and hsCRP matches with the meta-analysis by Li et al. [51], in which they demonstrated that serum hsCRP levels are higher in OSA patients compared with control subjects. CRP is primarily under regulation of IL-6 and is widely associated with cardiovascular diseases, since more than 20 large prospective studies suggest that hsCRP could be a predictor of future cardiovascular events [52]. However, patients with OSA have a number of confounding factors, such as obesity or diabetes, so increased CRP levels associated with OSA should be taken with precaution [53]. Several studies have attempted to link CRP with OSA independently from obesity, including that of Lui et al. where healthy middle-aged man had elevated CRP levels associated with OSA [54]. Moreover, according to Watanabe et al., UII may play a key role in the pathogenesis of atherosclerosis, jointly through hemodynamic effects and direct cellular actions on the vessel wall. It has been demonstrated that UII has a role in fatty streak formation, which is the initiation of atherosclerotic lesions [55]. Accumulating evidence has shown that UII is also closely linked to metabolic syndrome [56].

There are several limitations to this study. First of all, its cross-sectional design does not allow any causal conclusion, while also the sample size was relatively small and it was conducted in a single center. Furthermore, it was impossible to eliminate all of the possible confounding effects which could have interfered with the results. Lastly, full-night PSG was not performed in the control group among participants that obtained a low score on the STOP-Bang scale (<3). Therefore, it is impossible to fully exclude that some of the control subjects have had undetected OSA. However, a STOP-Bang score of 3 or less has a negative predictive value for OSA status and it is well established instrument for screening, as demonstrated in various validation analyses.

## 5. Conclusions

In conclusion, this study showed that serum UII levels are significantly higher in patients with OSA compared to healthy control subjects. Furthermore, there was a significant positive correlation with SBP, DBP, hsCRP, AHI and ODI. Therefore, given that urotensin serum concentrations were associated with blood pressure, but also indices of inflammation and OSA severity, we may infer that UII might play an important role in the complex pathophysiology of OSA. Yet, whether UII serum levels reflect metabolic and vascular changes in OSA, or UII itself acts as a mediator in its pathophysiology remains to be disclosed. Furthermore, in future perspectives it would be important to investigate UII interactions in CPAP-treated patients as well as the impact of treatment on the UII levels in these patients. Hence, future larger longitudinal studies are needed to further elaborate this subject.

## Figures and Tables

**Table 1:** Anthropometric and laboratory parameters of the study sample.

Parameter	Study Sample N = 178	OSA Group N = 89	Control Group N = 89	*p*
Age (years)	51.3 ± 8.4	52.5 ± 8.8	50.1 ± 7.9	0.056 ^*^
Male sex (N, %)	130 (73.0)	66 (74.2)	64 (71.9)	0.856 ^†^
Body height (cm)	184.1 ± 6.8	184.0 ± 7.0	184.2 ± 6.8	0.828 ^*^
Body weight (kg)	94.2 ± 12.9	95.3 ± 14.5	93.1 ± 11.2	0.245 ^*^
BMI (kg/m^2^)	27.7 ± 3.1	28.1 ± 3.4	27.4 ± 2.7	0.137 ^*^
Neck circumference (cm)	40.5 ± 2.6	42.7 ± 3.0	38.7 ± 2.6	<0.001 ^*^
SBP (mmHg)	132.0 ± 16.4	132.6 ± 15.9	131.3 ± 15.1	0.577 ^*^
DBP (mmHg)	83.8 ± 10.8	84.2 ± 9.4	83.1 ± 10.9	0.472 ^*^
LDL (mmol/L)	3.7 ± 0.6	3.8 ± 0.6	3.7 ± 0.7	0.517 ^*^
HDL (mmol/L)	1.39 ± 0.20	1.36 ± 0.19	1.41 ± 0.21	0.092 ^*^
Total cholesterol (mmol/L)	5.6 ± 1.2	5.6 ± 1.3	5.5 ± 1.2	0.732 ^*^
Triglycerides (mmol/L)	1.53 ± 0.35	1.57 ± 0.34	1.50 ± 0.35	0.200 ^*^
hsCRP (mg/L)	2.19 ± 0.97	2.87 ± 0.71	1.52 ± 0.68	<0.001 ^*^

All data are presented as whole number (percentage) or mean ± standard deviation. Abbreviations: BMI—body mass index; hsCRP—high-sensitivity C-reactive protein; SBP—systolic blood pressure; DBP—diastolic blood pressure; LDL—low-density lipoproteins; HDL—high-density lipoproteins. * Student’s *t*-test. ^†^ chi-square test.

**Table 2:** Polysomnographic parameters of the OSA group.

Parameter	OSA Group N = 89
AHI (events/h)	39.0 (31.4–55.2)
Mean SpO_2_ (%)	94.0 (92.3–95.0)
Lowest SpO_2_ (%)	76.4 (71.0–83.7)
ODI (events/h)	36.8 (27.8–56.1)
TST (hours)	6.3 (5.1–7.3)
Central apnea (total events)	15.5 (5.0–33.0)
Obstructive apnea (total events)	104.5 (47.5–168.5)
Mixed apnea (total events)	14.5 (6.5–39.5)
Hypopnea (total events)	92.5 (52.0–142.0)

All data are presented as median (interquartile range). Abbreviations: AHI—apnea/hypopnea index; ODI—oxygen desaturation index; TST—total sleep time.

**Table 3:** Urotensin II correlations with anthropometric and laboratory parameters in the whole study sample and the OSA group.

	Study Sample N = 178	OSA Group N = 89	Control Group N = 89
Parameter	r * *p*	r * *p*	r * *p*
Age (years)	0.087; 0.249	0.028; 0.793	0.151; 0.157
BMI (kg/m^2^)	0.176; 0.019	0.151; 0.156	0.192; 0.071
LDL (mmol/L)	0.069; 0.362	0.078; 0.463	0.060; 0.576
HDL (mmol/L)	−0.024; 0.819	−0.101; 0.180	−0.009; 0.933
Total cholesterol (mmol/L)	0.110; 0.143	0.376; <0.001	0.012; 0.911
Triglycerides (mmol/L)	0.032; 0.669	0.021; 0.842	0.043; 0.689
hsCRP (mg/L)	0.450; <0.001	0.413; <0.001	0.481; <0.001

Abbreviations: BMI—body mass index; hsCRP—high-sensitivity C-reactive protein; LDL—low-density lipoproteins; HDL—high-density lipoproteins. * Pearson’s correlation coefficient.

**Table 4:** Multiple linear regression model with serum urotensin II levels as a dependent variable.

Parameter	β *	SE ^†^	*t* Value	*p*
Age (years)	−0.002	0.012	−0.173	0.863
BMI (kg/m^2^)	0.017	0.034	0.515	0.608
SBP (mmHg)	0.052	0.008	6.305	<0.001
hsCRP (mg/L)	0.538	0.164	3.282	0.001
AHI (events/h)	0.017	0.006	2.532	0.013

Abbreviations: BMI—body mass index; hsCRP—high-sensitivity C-reactive protein; SBP—systolic blood pressure; DBP—diastolic blood pressure; AHI—apnea/hypopnea index; ODI—oxygen desaturation index. * unstandardized coefficient β. ^†^ standard error.

## Data Availability

The data presented in this study are available on request from the corresponding author. The data are not publicly available due to ethical restrictions.
